# Supplementary figures and images for: Role of T cells during the cerebral infection with Trypanosoma brucei
Source: PLoS Negl Trop Dis. 2021 Sep 29;15(9):e0009764. doi: 10.1371/journal.pntd.0009764 (PMC8530334; doi:10.1371/journal.pntd.0009764)

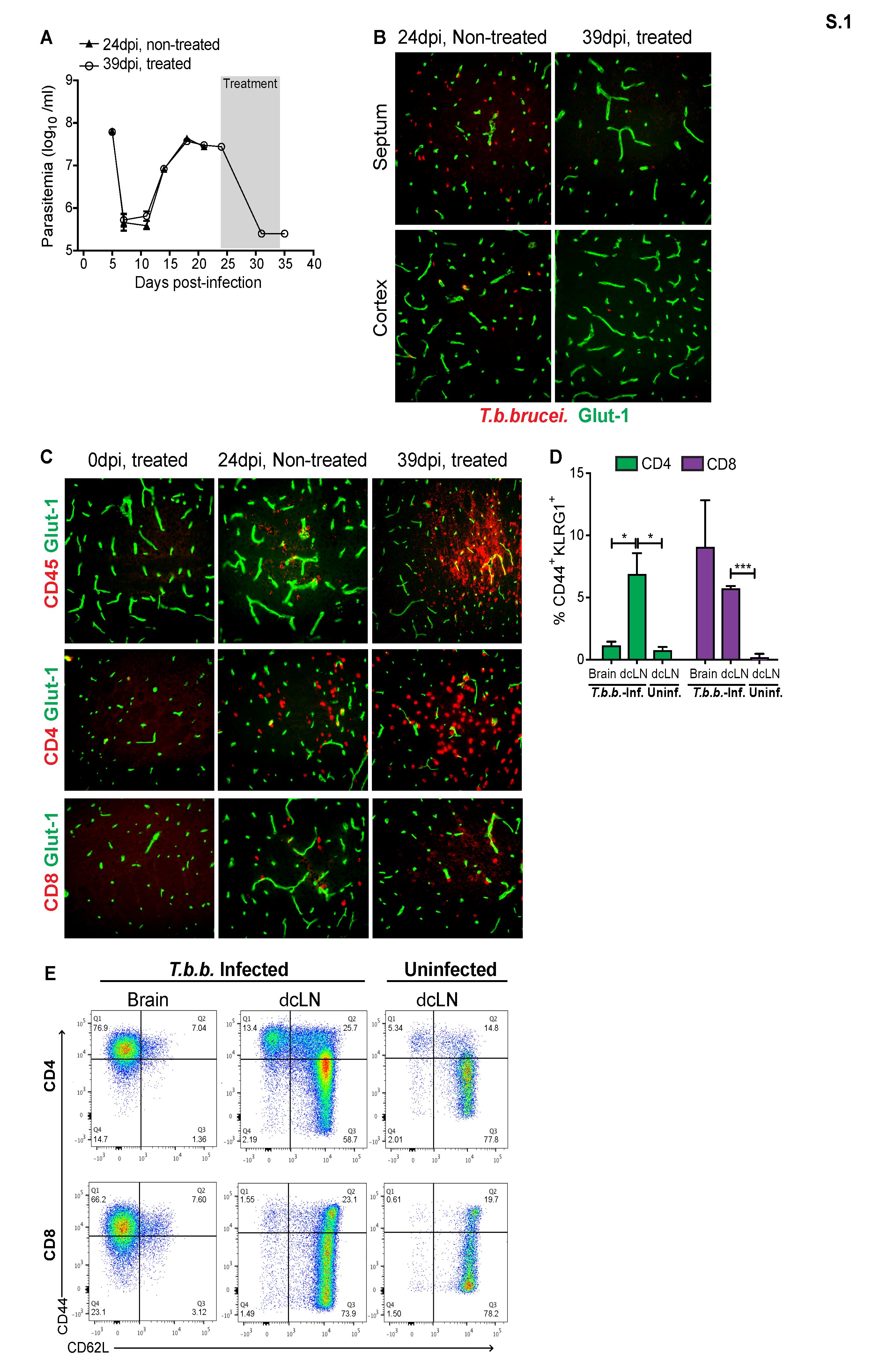

Supplement: S1 Fig — (A) The parasitemia of mice infected with T.b.b. and treated with melarsoprol and suramine starting at 21 dpi, is shown. (B) Micrographs comparing the presence of T.b.b. in the septum and cortex of untreated and suramin and melarsoprol treated mice at the indicated time points after infection. (C) Micrographs comparing the density of CD45+ leukocytes in the corpus callosum of mice infected or not with T.b.b. and treated with melarsoprol and suramin or untreated controls. (D) The mean % CD44+KRLG1+ CD4 and CD8 T cells ± SEM was measured in the brain and dcLN from either T.b.b.-infected (24 dpi) or control mice. Differences are significant at * p≤0.05 and ***p≤0.001 Student’s t test with Welch’s correction. (E) Representative dot plots showing the expression of CD44 and CD62L in the brain and dcLN CD4 and CD8 T cells from T.b.b.-infected and uninfected controls. (TIFF) [file pntd.0009764.s001.tiff]

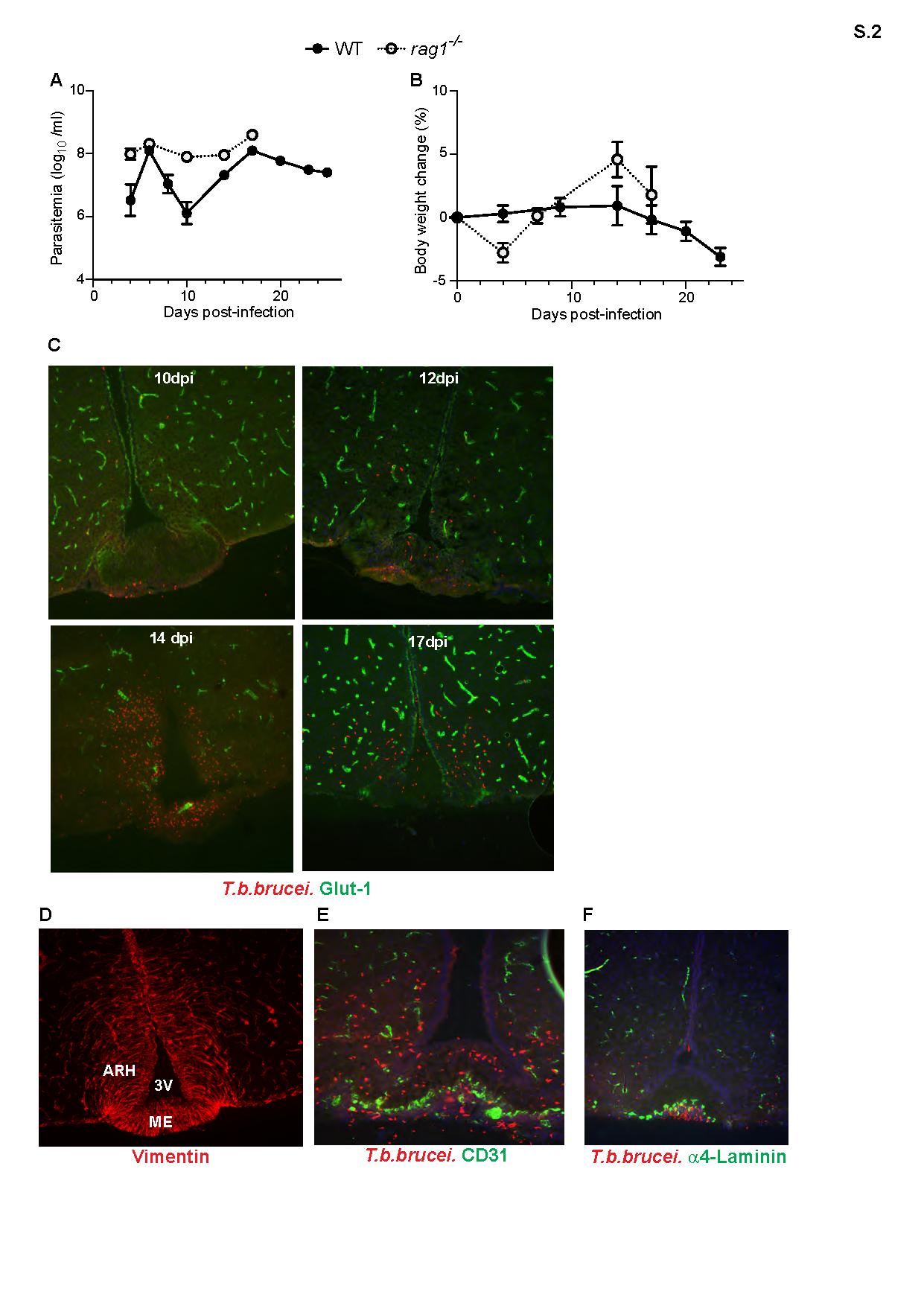

Supplement: S2 Fig — (A) The mean log10 parasitemia and (B) the percentage of weight change of WT and rag1-/- mice infected i.p. with 2000 T.b.b. (n ≥5 animals per group). (C) Micrographs showing the labeling of T.b.b. and glut1 in the ME of mice at different days after infection. Parasites were not detected in the ME of mice at 7 dpi. (D) Vimentin staining of tanycytes and other ependymal cells in the ME/ Arc. (E) Micrograph showing the extravascular localization of parasites in the ME brains of mice infected with T.b.b. at 14 dpi by staining with CD31. (F) Labeling of laminin and T.b.b. showing extravascular parasites in the ME. (TIFF) [file pntd.0009764.s002.tiff]

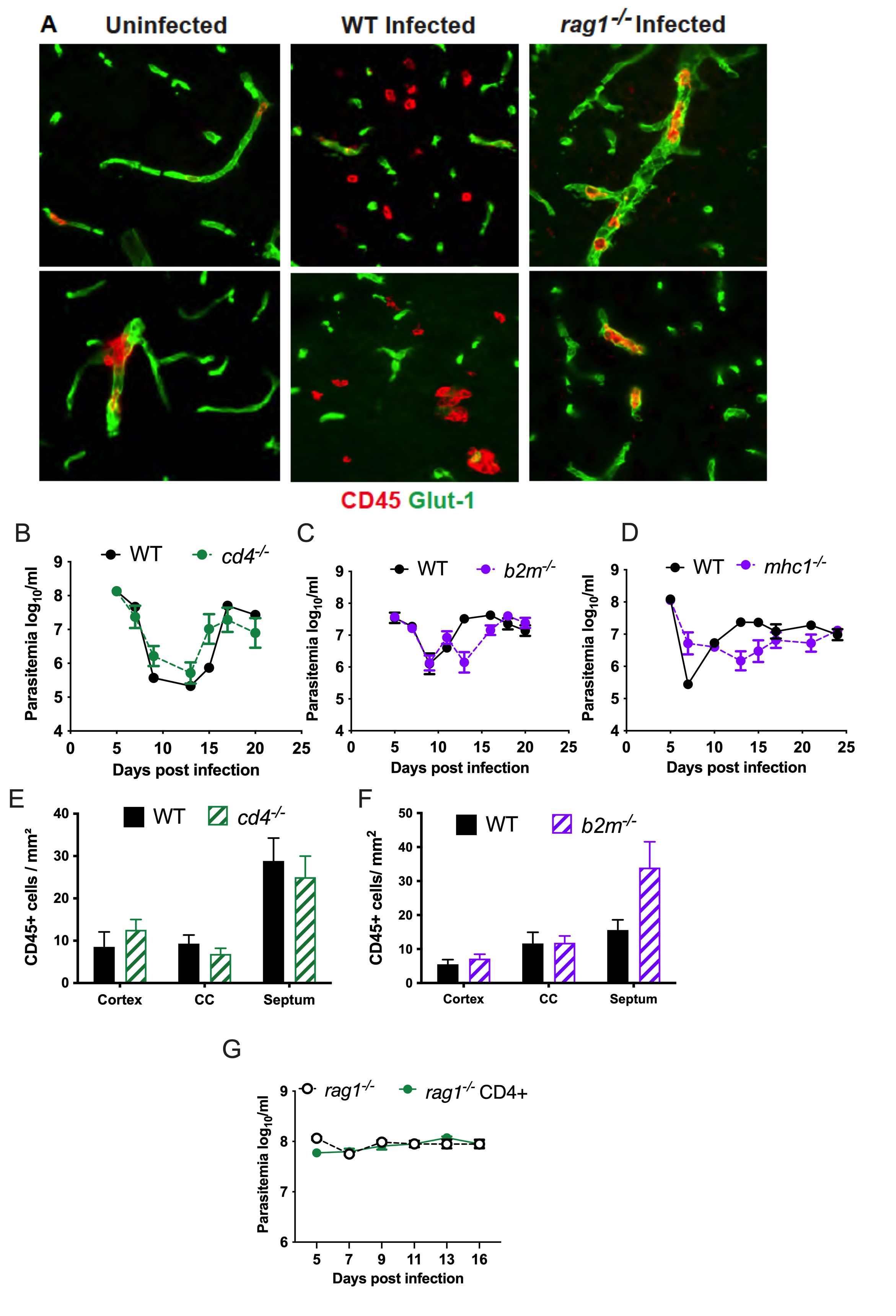

Supplement: S3 Fig — Micrographs showing the labeling for CD45 and glut1 in the cortex of WT and rag1-/- mice at 14 dpi with T.b.b. (B-D) The mean log10 parasites/ ml blood of WT and cd4-/- (B), b2m-/- (C) and mhc1-/- (D) and WT mice at different times after infection with T.b.b. (n ≥5 animals per group). (E, F) The mean number of CD45+ cells per mm2 in different brain regions of cd4-/ (E), b2m-/- (F) and WT mice at 23 dpi is shown. (G) The mean log10 parasites/ ml blood of CD4 T cell transferred and non-transferred rag1-/- mice at different times after infection with T.b.b. (n ≥5 animals per group). (TIFF) [file pntd.0009764.s003.tiff]
