# Supplementary material for: Role of T cells during the cerebral infection with Trypanosoma brucei
Source: PLoS Negl Trop Dis. 2021 Sep 29;15(9):e0009764. doi: 10.1371/journal.pntd.0009764 (PMC8530334; doi:10.1371/journal.pntd.0009764)
Supplement: S1 Table — (DOCX) [file pntd.0009764.s004.docx]

### S1 Table. Primary antibodies used for immunolabelling tissue sections

| **Primary ab** | **Species** | **Dilution** | **Source** |
| --- | --- | --- | --- |
| An Tat 1.1 VSG  *(T. brucei*) | rabbit | 5000 | Gift from P Buscher, ITG, Antwerp |
| CD4 | rat | 40 | BD 550278 |
| CD8 | rat | 40 | BD 553027 |
| CD45 | rat | 20 | BD 550539 |
| Glut-1 | goat | 40 | Santa Cruz Biotechnology Sc-1605 |
| ZO-1 | rabbit | 100 | Invitrogen 402200 |
| Meca32 | rat | 200 | Biolegend 120501 |
| CD31 | rat | 100 | BD 553370 |
| α4 laminin | rabbit | 500 | gift from K. Kristensson, KI |
| Vimentin | rabbit | 200 | Cells Signaling 5741 |
