# Supplementary material for: Role of T cells during the cerebral infection with Trypanosoma brucei
Source: PLoS Negl Trop Dis. 2021 Sep 29;15(9):e0009764. doi: 10.1371/journal.pntd.0009764 (PMC8530334; doi:10.1371/journal.pntd.0009764)
Supplement: S2 Table — (DOCX) [file pntd.0009764.s005.docx]

### S2 table. Primers used for RT-PCR

| **mRNA target** | **Forward** | **Reverse** |
| --- | --- | --- |
| *Meca32* | AATTGTAGATGAGCGTTC | TTATGTGCCAGTAATATCTT |
| *Vegfa* | TAGAGTACATCTTCAAGCCG | TCTTTCTTTGGTCTGCATTC |
| *Glut1* | AAGTCCAGGAGGATATTCAG | CTACAGTGTGGAGATAGGAG |
| *Il1b* | TGG TGT GTG ACG TTC CCA TT | CAG CAC GAG GCT TTT TTG TTG |
| *Hif1a* | CGATGACACAGAAACTGAAG | GAAGGTAAAGGAGACATTG |
| *Ifng* | GCT TTG CAG CTC TTC CTC AT | CAC ATC TAT GCC ACT TGA GTT AAA ATA GT |
| *Tnf* | GGC TGC CCC GAC TAC GT | GAC TTT CTC CTG GTA TGA GAT AGC AAA |
| *Hprt* | CCC AGC GTC GTG ATT AGC | GGA ATA AAC ACT TTT TCC AAA TCC |
