# Supplementary material for: Role of T cells during the cerebral infection with Trypanosoma brucei
Source: PLoS Negl Trop Dis. 2021 Sep 29;15(9):e0009764. doi: 10.1371/journal.pntd.0009764 (PMC8530334; doi:10.1371/journal.pntd.0009764)
Supplement: Attachment — Submitted filename: Cover letter reviewed.pdf [file pntd.0009764.s006.pdf]

Department of Microbiology, Tumor and Cell Biology

Martin Rottenberg, Professor

Stockholm, 10 July 2021

Dr Mary Ann McDowell and Michael Boshart,
Editors,
PLOS Neglected Tropical Diseases

Dear Dr Mc Dowell and Boshart,

Please find uploaded the electronic version of our re-revised manuscript "Role of T cells during the cerebral infection with *Trypanosoma brucei*" PNTD-D-21-00411 by Olivera et al. We have uploaded two versions of the revised manuscript: one with either highlights or tracked changes denoting where the text has been changed; the other a clean version (uploaded as the manuscript file) as requested.
Thank you for the opportunity to respond to the comments from the reviewers. Below, we have responded to each point and indicated where modifications to the manuscript were made. Our responses to their suggestions and the changes made are marked in blue. We believe that this revision has resulted in an improved manuscript.

Reviewer 1:

Olivera et al. studied the kinetics, functional status and anatomic location of CNS T cells in murine Trypanosoma brucei brucei infection.
Based on their results the authors conclude that early upon systemic infection of mice with Tbb effector-memory T cells but at later stages resident memory T cells are present in the brain. T cells control Tbb but are also required for invasion of Tbb into the brain parenchyma. These data provide significant new information on the relation of T cells, brain invasion and spread of T.b.b., and clinical signs of sleeping disease in mice.
The authors used a broad spectrum of techniques including sophisticated mouse models to unravel the T cell-specific immune response in different compartments of the CNS during T.b.b. infection. Data are well presented in the figures, which have a high quality, and interpretations are plausible.
However, some important points remain, in particular to readability of the manuscript as detailed below.

Specific comments:
- For readers not familiar with the different anatomic structures of the CNS, i.e. the majority of PNTD readers, the manuscript is difficult to understand. E.g. in the Abstract the authors state that T cells are required for brain invasion of T.b.b. to the brain. Some few sentences later the authors report that parasite numbers were

higher in the median eminence, a brain structure “with increased vascular permeability”, of rag-/- mice. WT mice.

To avoid confusion for those readers not familiar with the different anatomic structures and the BBB it would be helpful, if the authors carefully revise the manuscript and provide better information on the different anatomic structures of the CNS and their immunological differences.

We thank for the reviewer for this comment. We provided a graphical abstract in which readers can be oriented on the anatomical structures of the CNS and provided information in the legend to that figure (Figure 6 panel G).

- In the same line, lines 209-217 need more explanations: what are tanycytes? Why do the authors stain for glucose transporters?

We have explained in the legend to the graphical abstract the role of tanycytes.

It reads like the ME is not part of the brain parenchyma? Correct?

- Eventually a graphical illustration demonstrating T cells and T.b.b. in different CNS compartments over time would be helpful.

To address the comment from the reviewer, we have added a graphical abstract / sketching the location of the brain anatomical structures and the BBB, as well as the location of *T.b.b.* and T cells at early and late stages after *T.b.b.* infection (Figure 6 G). In this figure we show the medium eminence, located at the base of the hypothalamus, where capillaries are highly fenestrated with less tight junctions between endothelial cells creating a more permeable barrier. In the median eminence, (ME) the barrier has been displaced to the walls lining the 3rd ventricle and to the interphase between the ME and the Arc. Here the tanycytes, specialized radial glia cells, form a physical barrier to control the correct transport of nutrients and metabolic hormones into the brain parenchyma.

During the early systemic infection, *T.b.b.* parasites and leukocytes move from the vascular layer at the bottom of the ME into the floor of the 3rd ventricle and into the Arc. We show that T cells facilitate the passage of other leukocytes into the lumen of the ME and the Arc but hamper parasite infection. T cells, other inflammatory cells and parasites stimulate the production of metabolic and inflammatory molecules that increase the permeability of blood hypothalamus barrier. Further T cell activity regulate sleep wake rhythm during Tbb infection. At this early-stage T cells in the brain differentiate into a memory phenotype (T_{EM}), and low levels of these can be found in the brain parenchyma.

In a second stage of infection T cells and *T.b.b.* invade the brain parenchyma by breaching the BBB. Both CD4 and CD8 T cells can independently of each other penetrate the brain parenchyma. A fraction of T cells in the brain will also differentiate into resident memory phenotype (T_{RM}). We speculate that T_{RM} secrete chemokines that attract vascular T cells to the brain parenchyma. We have included this sketch

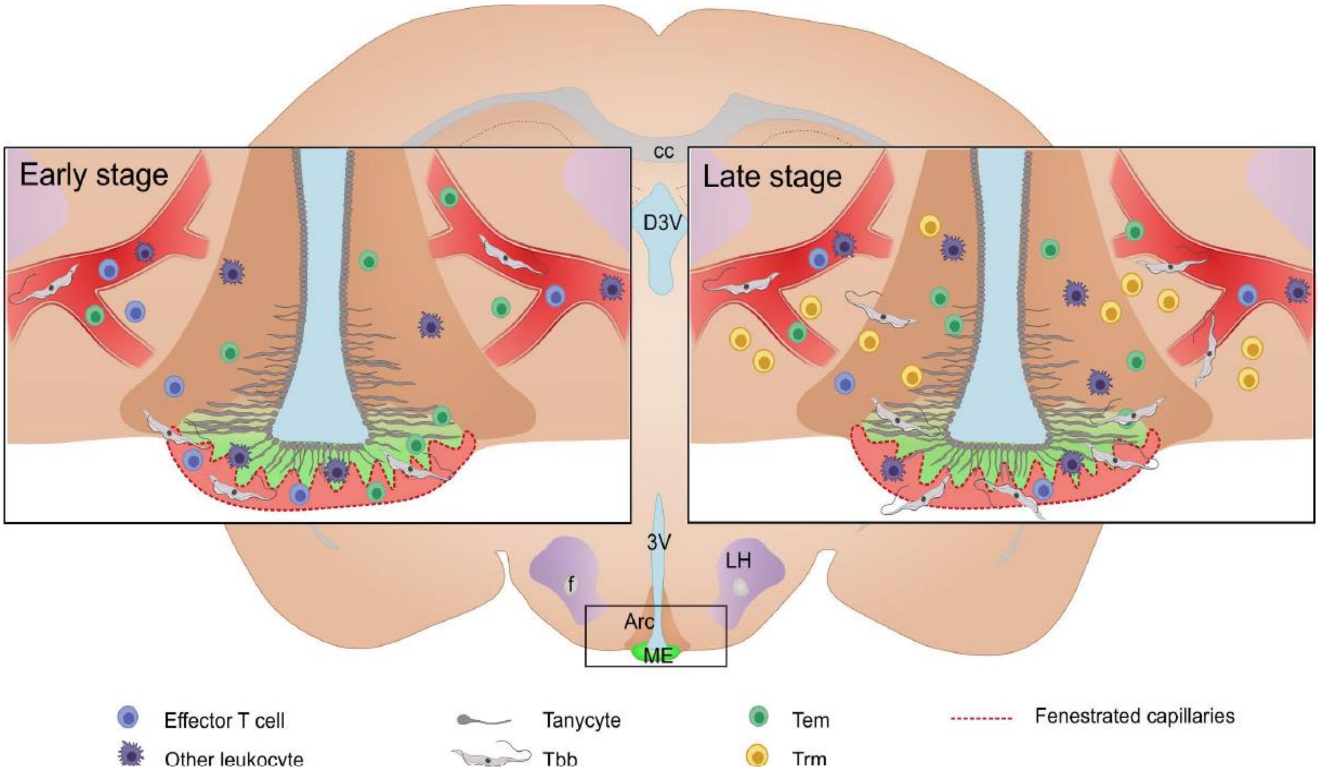

the anatomical localization and part of our results below as Figure 6 panel G in our revised manuscript.

- Additionally, the Abstract does not cover several relevant aspects of the study including the experiments with CD4-/- mice. Please, refine the Abstract.

We have added into the abstract of our revised manuscript the information provided by experiments using mice deficient in CD4 and CD8 T cells.

- Also lines 389-400: what is sleep/wake transitions and sleep bouts? Please provide better explanation.

Sleep bout is the period spent sleeping. Sleep/ wake transitions are the number of transitions between sleep and wakefulness or between wakefulness and sleep.

- Line 404: point to – before or during?

- CD4 T cell outnumber CD8 T cells by far in Tbb-infected WT mice. However, in Tbb-infected WT and CD4-/- mice, numbers of intracerebral CD8 T cells did not differ and IFNg levels were equal. In this context, the authors should discuss that the CD8 T cell compartment of CD4-/- contains MHC cl. II-restricted CD8 T cells and reduced numbers of MHC cl. I-restricted CD8 T cells and how this different MHC restriction of CD8 T cells of CD4-/- mice may affect the T cell response, pathogen control etc. Eg. See interpretation of data lines 256-257, 362-369.

We thank the reviewer for this comment, which was also brought up by reviewer 2. In the original manuscript we showed that the deficiency of CD4 T cells

neither results in altered invasion of parasites and leukocytes into the ME/ Arc nor changes the levels of penetration of T cells and parasites into the brain parenchyma. Following the suggestion of the reviewers we have performed studies using mice genomically lacking either MHC-I or the beta-2-microglobulin (*mhc1*$^{-/-}$ and *b2m*$^{-/-}$ mice) and thereby devoid of mature CD8 T cells. Neither the penetration of leukocytes and parasites into the brain parenchyma, nor the parasitemia levels of *b2m*$^{-/-}$ or *mhc1*$^{-/-}$ mice were different to those displayed by WT controls. Thus, both CD4 and CD8 T cells can independently invade and promote parasite penetration into the brain during the late phase of infection. In other words, neither CD4 nor CD8 T cells are required for the invasion by T cells and parasites.

Cd4$^{-/-}$ mice showed no reduction of *inos, tnf* and *ifng* transcripts coding for inflammatory molecules that are required for brain invasion by T cells and *Tbb*. This suggests that either CD4 T cells play no role in the production of these inflammatory molecules or that in *cd4*$^{-/-}$ mice there is a compensation whereby other immune cell populations can drive the production of cytokines that are otherwise produced by CD4 T cells. To address the comment of reviewer 1, we have inoculated T and B cell deficient rag1$^{-/-}$ mice with 10^6 sorted CD4 T cells. Three weeks after CD4 T cell transfer, mice were infected with *T.b.b*. We observed, as expected, the presence of CD4 but not CD8 T cells in blood and spleens from infected mice *rag1*$^{-/-}$ transferred with CD4 T cells. *Rag1*$^{-/-}$ mice transferred with CD4 T cells (but not non-transferred controls) showed *T.b.b.* in different brain regions. Moreover, brains from *rag1*$^{-/-}$ mice transferred with CD4 T cells showed higher expression of *ifng, inos* and *tnf* mRNA than the non-transferred controls. Altogether, our data suggest that neither CD4 nor CD8 T cells are required for penetration of leukocytes or parasites into the brain. Both CD4 and CD8 T cells can penetrate the brain parenchyma even in the absence of the other population. On the other hand, CD4 T cells were sufficient to invade the brain and to induce the production of inflammatory molecules required for the penetration of inflammatory cells and parasites. We have added these new results as a new Figure 4 and in the supplementary figure 3 in our revised manuscript, as well as included these data in the abstract, results and discussion sections.

As suggested by the reviewer, compensations in the genetically deficient *cd4*$^{-/-}$ mice during infection may also have an impact in the results obtained. The numbers of CD8 in the thymus were normal but were expanded in the periphery. In fact the numbers of T cells in these lymph nodes from cd4^{--} have been shown to be similar to those of WT controls, with 90% of peripheral $\alpha\beta$ T cells were CD8 T cells [1]. Moreover, MHC-II-restricted, double negative TCR$\alpha\beta^{+}$ CD4-CD8-T cells have been shown to develop in *cd4*$^{-/-}$ mice [2], and as indicated by the reviewer the CD8 T cell population of cd4$^{-/-}$ mice was shown to be contamined with MHC-II restricted cells [3].

Despite these compensations, T cell help for the production of antibodies [1], and mounting and maintaining proper CD8 T cell cytotoxicity were reduced [4]. CD4$^{-/--}$ were shown to be susceptible to infection with *Toxoplasma gondii [5], Trypanosoma* cruzi [6], *Mycobacterium tuberculosis [7]*, LCMV [8] and *Listeria monocytogenes* [4] indicating a non-redundant role of CD4 T cells in these mice. This was addressed up as suggested by the reviewer in the discussion section of our revised manuscript.

Reviewer #2: In this work, the authors characterize the T cell compartment in the brain in comparison with deep cervical lymph nodes in mice infected with Trypanosoma brucei brucei (T.b.b) at several points of infection and with

trypanocidal treatment. They further phenotypically characterize the T resident memory cells based on PD-1 expression. They confirm previous results showing that T cells are required for T.b.b. invasion of brain parenchyma but here they discard the involvement of CD4 T cells. They further show that there is no difference between the expression of factors related to endothelial cell function and inflammation between WT and Rag1 KO mice. Besides differences in IFNg production, Rag1 KO mice show less sleep alterations compared to WT mice.
This study shows some interesting findings such as the fact that CD4 T cells are not required for parasite or leukocyte invasion of the parenchyma and for IFNg production, and the role of lymphocytes in sleep alterations. However several findings are poorly characterized and connected between figures. Moreover, the discussion does not mention the role of other lymphocytes besides CD4 T cells in the brain, namely of CD8 T cells:
1) In Fig. 1 the authors show that trypanocidal treatment does not affect the number of T cells in the brain although they do not further characterize them based on PD1 expression.

We have measured the expression of PD1 in T cells from WT infected with *T.b.b.* before and after treatment with melarsoprol. CD4 T cells from the brain of infected mice showed a reduced frequency of expression of PD1 as compared to those before treatment. Instead, the frequency of PD1 expression in CD8 T cells from infected mice before and after infection with *T.b.b.* was similar.

Furthermore, they do not show whether in treated mice there are alterations in the expression of inflammatory cytokines as done for Rag1 KO mice. Also, CCR7 expression used to distinguish central memory from effector memory cells is not shown here. In the Discussion, the authors mention low effector function of these cells but they have not performed any functional assay or have used markers of proliferation.

Thank you for this comment. We have used CD44 marker to define the activated/ memory T cells and KRLG1 expression to define the short lived effector cells (so KRLG1- cells are long lived non-effector=memory populations) and the expression of CD62L to define lymph node resident vs circulating memory T cells. We agree with the reviewer that other markers can be used to define T_{EM} and T_{CM} populations. For example, T_{EM} can be defined by CD62^{lo}/ CCR7^{lo}/ CD27^{lo}/ CD127^{hi}/ Blimp1^{hi} and ID2^{hi}, whereas T_{CM} subset can be characterized by CCR7^{hi}/ CD62L^{hi}/ CxCr1^{lo}/CD27^{lo}/CD127 ^{hi}/Eomes^{hi}/ ID3^{hi}/ BCL6 ^{hi} [9]. The expression of some of these molecules may vary with time and antigen-encounter and are used to capture (in part) the phenotypic heterogeneity within the memory subset. Thus, we believe that our characterization of T cell memory subsets is appropriate, although we omit part of the diversity of memory T cell functions.

2) In Fig. 3 the authors demonstrate that CD4 T cells are not required for parasite or leukocyte invasion and for IFNg induction. This strongly suggests that CD8 T cells may play a fundamental role in the brain in infected mice. Did the authors try to treat WT mice with anti-CD8? The potential role of CD8 T cell in the infection should at least be addressed in the discussion.

This question was also addressed in a detailed form in the responses to reviewer 1 above. As indicated, we have included in the revised manuscript the comparison of *T.b.b.* infection in 2 different mouse strains (*mhc1*$^{-/-}$ *and b2m*$^{-/-}$) that lack CD8 T cells. We have also included experiments in which *rag1*$^{-/-}$ mice were reconstituted with CD4 T cells to address these comments. These data suggest that both CD4 and CD8 T cells are redundant for penetration of either leukocytes or parasites into the brain. In other words, CD4 and CD8 T cells can penetrate the brain parenchyma even in the absence of the other population. On the other hand, CD4T cells were sufficient to invade the brain and to induce the production of inflammatory molecules required for the penetration of inflammatory cells and parasites. As suggested by the reviewer, compensations in the genetically deficient *cd4*$^{-/-}$ mice during infection may also have an impact in the results obtained.
We have added these new results as a new Figure 4 and modified the supplementary figure 3 in our revised manuscript, as well as included these data in the abstract, results and discussion sections.

3) In Fig. 4 the authors show that in Rag1 KO mice, besides differences in IFNg, the expression of other metabolism or inflammation-related genes is not affected. Is the expression of these genes dependent on IFNg? Why the authors are showing day 27 of infection?

The expression of the other transcripts measured in Fig 4 is not regulated by IFN-γ. We have added the analysis of WT mice at 27 days since these are undergoing the late stage of infection. The comparison with rag1$^{-/-}$ mice could not be included since in this model *rag1*$^{-/-}$ mice only survived for ca 20-22 days after infection.

4) In Fig. 5 although the authors find differences in sleep patterns between WT and Rag1 KO mice, it would be interesting to know whether they are related to an altered function of the hypothalamus such as production of orexin/hypocretin.

Peptidergic neurons in the lateral hypothalamus containing orexin and melanin-concentrating hormone (mch) have been shown to be reduced and suggesting their involvement in the pathogenesis of sleep/wake alterations in sleeping sickness [10]. We have performed in situ hybridization experiments labelling *npy* (neuropeptide y) and *mch* (transcripts in the brain in WT and *rag1*$^{-/-}$ mice sacrificed at the start of the light period, infected or not with *T.b.b.* Variation on our results precluded a clear interpretation of these data, so we these were omitted. We also agree with the reviewer that to understand the regulation of sleep alterations by T cells during *T.b.b.* infection.

Other issues:
1) Legend of Figure 1 does not mention K.
We apologize for this mistake, and we thank the reviewer for pointing it out. We have corrected the labeling of several panels in the legend to Figure 1.

2) In line 158, “The levels of both TEM and TCM in the dcLN were increased after

…" but the graph shows that an increase is only significant for TEM in case of CD4 T cells and TCM in case of CD8 T cells.

We acknowledge the reviewer for this comment. We have corrected this mistake in the text of the results section from our revised manuscript.

We appreciate the time and effort of the reviewers, and the opportunity to respond to their suggestions. We have revised the manuscript where indicated above, and would appreciate if the revised manuscript is considered acceptable for publication in the **PLOS Neglected Tropical Diseases**.

Sincerely Yours,

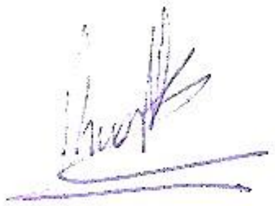

1. Rahemtulla A, Fung-Leung WP, Schilham MW, Kundig TM, Sambhara SR, Narendran A, Arabian A, Wakeham A, Paige CJ, Zinkernagel RM, et al. Normal development and function of CD8+ cells but markedly decreased helper cell activity in mice lacking CD4. Nature. 1991;353(6340):180-4.10.1038/353180a0
2. Rahemtulla A, Kundig TM, Narendran A, Bachmann MF, Julius M, Paige CJ, Ohashi PS, Zinkernagel RM, Mak TW. Class II major histocompatibility complex-restricted T cell function in CD4-deficient mice. Eur J Immunol. 1994;24(9):2213-8.10.1002/eji.1830240942
3. Tyznik AJ, Sun JC, Bevan MJ. The CD8 population in CD4-deficient mice is heavily contaminated with MHC class II-restricted T cells. J Exp Med. 2004;199(4):559-65.10.1084/jem.20031961
4. Sun JC, Bevan MJ. Defective CD8 T cell memory following acute infection without CD4 T cell help. Science. 2003;300(5617):339-42.10.1126/science.1083317
5. Johnson LL, Sayles PC. Deficient humoral responses underlie susceptibility to Toxoplasma gondii in CD4-deficient mice. Infect Immun. 2002;70(1):185-91.10.1128/IAI.70.1.185-191.2002
6. Rottenberg ME, Bakhiet M, Olsson T, Kristensson K, Mak T, Wigzell H, Orn A. Differential susceptibilities of mice genomically deleted of CD4 and CD8 to infections with Trypanosoma cruzi or Trypanosoma brucei. Infect Immun. 1993;61(12):5129-33.10.1128/iai.61.12.5129-5133.1993
7. Caruso AM, Serbina N, Klein E, Triebold K, Bloom BR, Flynn JL. Mice deficient in CD4 T cells have only transiently diminished levels of IFN-gamma, yet succumb to tuberculosis. J Immunol. 1999;162(9):5407-16

8. Rahemtulla A, Shahinian A, Kundig T, Zinkernagel R, Mak TW. CD4 negative mice as a model for immunodeficiency. Philos Trans R Soc Lond B Biol Sci. 1993;342(1299):57-8.10.1098/rstb.1993.0135

9. Martin MD, Badovinac VP. Defining Memory CD8 T Cell. Front Immunol. 2018;9:2692.10.3389/fimmu.2018.02692

10. Palomba M, Seke-Etet PF, Laperchia C, Tiberio L, Xu YZ, Colavito V, Grassi-Zucconi G, Bentivoglio M. Alterations of orexinergic and melanin-concentrating hormone neurons in experimental sleeping sickness. Neuroscience. 2015;290:185-95.10.1016/j.neuroscience.2014.12.066
